# Dual Pathways From Self-Compassion and Self-Criticism to Academic Achievement: The Roles of Engagement and Exhaustion

**DOI:** 10.5334/pme.1821

**Published:** 2026-02-17

**Authors:** Piermarco Consiglio, Marjon Fokkens-Bruinsma, Marie-Christine Opdenakker, Ellen P. W. A. Jansen, Joke Fleer

**Affiliations:** 1 Department of Health Sciences, Section Health Psychology, University Medical Center Groningen, University of Groningen, The Netherlands; 2 Department of Teacher Education, University of Groningen, The Netherlands; 3 University College Groningen & Honours College, the Netherlands

## Abstract

**Background:**

Engagement and burnout symptoms among medical students are key factors influencing their academic performance, risk of dropout, and overall well-being. While research has primarily focused on negative constructs such as burnout symptoms, less attention has been given to how positive constructs, such as engagement, evolve over time. This study examines the temporal changes in medical students’ engagement and exhaustion while exploring two distinct pathways: the ‘bright path’, which investigates engagement as a mediator between self-compassion and academic achievement, and the ‘dark path’, which examines exhaustion as a mediator between self-criticism and academic achievement.

**Methods:**

Self-report measures were used to assess self-compassion, self-criticism, engagement, and exhaustion, while academic achievement was measured objectively. The data were drawn from a longitudinal research project that followed 117 medical students throughout their entire Bachelor’s program. Analyses included linear growth models and parallel process latent growth curve models to examine changes over time and potential mediation effects.

**Results:**

The findings show a decline in engagement and exhaustion throughout the Bachelor’s program. Furthermore, neither engagement nor exhaustion mediated the relationships within the bright and dark pathways.

**Conclusions:**

Findings underscore the importance of fostering environments that promote well-being and engagement while addressing negative factors like exhaustion that can hinder student success. They also highlight the potential influence of contextual factors in shaping medical students’ experiences, suggesting that interactions between personal resources and environmental demands may play a key role in shaping students’ engagement, exhaustion and academic achievement.

## 1. Introduction

High risk of burnout symptoms is a well-documented challenge in higher education, including medical programs [20,34,49,58,87]. Burnout symptoms can have negative consequences for students, such as decreased well-being and impaired academic performance [28,38]. Conversely, previous research highlights the importance of engagement – the positive antipode of burnout [51] – in fostering academic success, enhancing resilience, and reducing dissatisfaction and dropout rate [14,21–22,64,83]. Building on these insights, scholars increasingly emphasize the need of addressing not only negative constructs like burnout symptoms, but also positive constructs such as engagement [1,23,50].

However, previous research has focused primarily on burnout symptoms, often overlooking the construct of engagement. In particular, there is a lack of longitudinal research on engagement among medical students. Current evidence indicates that while the risk of experiencing burnout symptoms increases as medical students progress through their studies [11,30,36,39,66,79], it remains unclear how engagement changes over time. Therefore, in the current study, we focus on how both burnout symptoms and engagement change over time.

### 1.1 Job Demands–Resources Model and Study Hypotheses

Based on the Job Demands-Resources Model (JD-R) [4], we assume that the trajectories of burnout symptoms and engagement have an independent course. In fact, the JD-R model highlights burnout and engagement as distinct constructs encompassed within two key processes: (1) A motivational process influenced by the availability of job and personal resources, resulting in favourable outcomes (e.g., improved academic achievement, increased intrinsic motivation and greater resilience) through engagement, and, (2) A health impairment process initiated by job and personal demands, leading to adverse outcomes (e.g., diminished academic achievement) via burnout symptoms.

Based on this framework, self-compassion and self-criticism were selected as distinct predictors in the current study because they represent theoretically separate mechanisms: self-compassion serves as a personal resource supporting the motivational process, while self-criticism functions as a personal demand driving the health impairment process. Engagement and exhaustion serve as mediators in these respective pathways, allowing us to test how self-compassion and self-criticism influence academic achievement over time.

Building on these principles, the present study proposes the following hypotheses:

H1: Exhaustion and engagement change over the course of the Bachelor programme.

H2: Self-compassion predicts academic achievement indirectly through engagement.

H3: Self-criticism predicts academic achievement indirectly through exhaustion.

The focus of this study is therefore on self-compassion as a personal resource and self-criticism as a personal demand. Personal resources are aspects of the self that are generally linked to resiliency and influence individuals’ abilities to control and impact their environment successfully [32]. Research among university students explored the motivational process outlined in the JD-R model and found that personal resources, such as mindfulness, psychological flexibility, and self-efficacy, enhance student engagement [56,62]. Recently, self-compassion has emerged as a key personal resource within the JD-R model. It involves treating oneself with kindness and understanding during times of difficulty, failure, or suffering [54]. Research has shown a significant positive relationship between self-compassion and student engagement at the university level [46]. Additionally, self-compassion has been linked to improved mental health across various populations, including students [8,90]. By fostering positive physiological and psychological responses to stress, self-compassion helps students navigate challenging circumstances [7,48]. Through a kinder internal dialogue, self-compassion enhances emotional resilience and overall well-being [24].

As opposed to personal resources, personal demands are self-imposed expectations for performance and behaviour that require effort and can result in physical and psychological strain [5].

Previous research investigated the health impairment process outlined in the JD-R model by including personal demands as predictors. For example, [27] found that workaholism, which is the tendency to work excessively hard and being obsessed with work [68], acts as a personal demand that influences job demands and, in turn, increases the risk of experiencing burnout symptoms among employees. Likewise, recent studies among university students found that personal demands (e.g., irrational performance expectations, awfulizing and need for control) are linked to a higher risk of experiencing burnout symptoms [80–81]. As self-compassion has been identified as a personal resource in the JD-R model [46], in the current study, we argue that self-criticism is a personal demand. Self-criticism, defined as a harsh and judgmental attitude toward oneself in response to personal failures, mistakes, or shortcomings [54], is a key psychological construct. It is closely associated with various mental health challenges, including depression, anxiety, and eating disorders [82,85].

Beyond these psychological resources and demands, sociodemographic factors such as gender and nationality may also influence students’ mental health and academic functioning, and therefore warrant consideration as covariates in the present study. Studies assessing gender differences in burnout symptoms have yielded mixed results: some found that males are at greater risk of burnout symptoms [e.g., 2,19], while others suggest females report higher levels of engagement [65,78], which may be linked to their stronger academic performance [59]. Regarding nationality, international students face unique challenges that may impact their well-being and academic outcomes, including language barriers, homesickness, social isolation, and financial strain [55]. While some studies report no significant differences in mental health outcomes between domestic and international students [e.g., 41,44–45], others identify notable disparities in psychological distress, depression, and anxiety [18,40]. Given these contrasting findings, considering gender and nationality as covariates is important to ensure that observed associations among psychological variables and academic outcomes are not confounded by these background characteristics.

In this study, we examine two distinct pathways within the JD-R framework: the motivational process, which we call the “bright path”, explores how the trajectory of self-compassion is associated with the trajectory of academic achievement, with engagement serving as a mediating factor. In contrast, the health impairment process, which we call the “dark path”, investigates how the trajectory of self-criticism is associated with the trajectory of academic achievement, with exhaustion serving as a mediator. We use exhaustion as a proxy for burnout, as it is often considered its core component [52,70]. By incorporating academic achievement as an objective outcome within both the bright and dark pathways, this study contributes to the JD-R model by enhancing its practical applicability to educational contexts. This is noteworthy because among employees it has been shown that it is essential for the advancement of organizational psychology to incorporate into research models, such as the JD-R framework, objective measures that are directly relevant to business outcomes [4]. Similarly, incorporating objective outcomes into research using the JD-R model with medical students can enhance the relevance and applicability of findings to real-world educational settings, thereby increasing the practical value of the research.

In this study, we address the following research questions:

1. How do medical students’ exhaustion and engagement change over time during the three-year medical bachelor’s degree program?
2. What is the mediating role of engagement in the relationship between self-compassion and academic achievement over the course of the medical bachelor’s degree program?
3. What is the mediating role of exhaustion in the relationship between self-criticism and academic achievement during the medical bachelor’s degree program?

## 2. Methods

### 2.1. Design

This longitudinal study was conducted at a faculty of medical sciences in the Netherlands and followed two cohorts of students who started their bachelor’s programme in the academic years 2015/16 and 2016/17. Data were collected at six time points, with one-year intervals between measurements, covering both undergraduate and master’s programmes. The first cohort was followed from 2015 to 2020, and the second cohort from 2017 to 2022. For this study, the two cohorts were pooled. However, only data from the first three time points, corresponding to the bachelor’s years, were analyzed in this study. Medical students were recruited during class hours. A member of the research team presented the study to the students, who were then provided with an information packet containing a letter and an informed consent form. Participants gave informed consent after reading the study procedure and their participation rights. Participation was voluntary, and anonymity and confidentiality standards were assured for all participants. There were no inclusion or exclusion criteria, except that the participants had to be first-year medical students enrolled in the Bachelor’s program.

To track participants across the longitudinal study while maintaining anonymity, a unique code was assigned to each student. This code, which did not directly reveal the students’ identities, enabled matching and tracking of individual participants across each data collection wave. The study received ethical approval from the Nederlandse Vereniging voor Medisch Onderwijs Ethical Review Board (NVMO ERB) [Dutch Association for Medical Education Ethical Review Board] with the approval code 442.

### 2.2. Measures

**Self-compassion and self-criticism**. Self-compassion and self-criticism were measured using the Self-Compassion Scale Short Form (SCS-SF) [60]. This questionnaire consists of three subscales assessing self-compassion: Self-kindness (e.g., “I try to be understanding and patient towards those aspects of my personality I don’t like.”), Mindfulness (e.g., “When something painful happens I try to take a balanced view of the situation.”), and Common Humanity (e.g., “I try to see my failings as part of the human condition.”). In addition, three subscales assess self-criticism: Self-judgment (e.g., “I’m disapproving and judgmental about my own flaws and inadequacies.”), Over-Identification (e.g., “When I fail at something important to me, I become consumed by feelings of inadequacy.”), and Isolation (e.g., “When I’m feeling down, I tend to feel like most other people are probably happier than I am.”). Each subscale has two items, which are scored on a scale ranging from 1 (Almost never) to 5 (Almost always). As suggested by [42], the Self-kindness, mindfulness, and common humanity subscales were averaged to calculate a composite score. Similarly, a single composite score for self-criticism was calculated by averaging the self-judgment, over-identification, and isolation subscales. In the current study, Cronbach’s alphas were .70 for self-compassion at T1, .72 at T2, .76 at T3. For self-criticism, the Cronbach’s alphas were .83 at T1, .87 at T2, .88 at T3.

**Engagement**. To assess engagement, the Utrecht Work Engagement Scale for Students (UWES–9S) was used [67]. This is a scale comprising 9 items scored on a seven–point frequency rating scale ranging from 0 (*never*) to 6 (*always*). An overall engagement score was calculated by averaging the individual item responses. An example item is: “When I’m doing my work as a student, I feel bursting with energy”. In the present study, Cronbach’s alphas were .86 at T1, .89 at T2, .90 at T3.

**Exhaustion**. Exhaustion was assessed using the exhaustion subscale of the Maslach Burnout Inventory – Student Survey [69]. It consists of 5 items scored on a seven-point frequency rating scale ranging from 0 (Never) to 6 (Always). The scores of the items were then used to calculate an overall exhaustion score by averaging the individual item responses. An example items is: “I feel emotionally drained by my studies”. In this study, the Cronbach’s alphas for exhaustion were .83 at T1, .88 at T2 and T3.

**Academic achievement**. To assess academic achievement, the grades from all exams taken by the students during each academic year were aggregated and averaged to compute a composite score for each year. Scores in the Dutch university system range from 1 to 10, with 1 representing the lowest grade and 10 the highest, and a score of 5.5 representing the minimum passing grade. This data was obtained from university records at the end of each academic year.

**Socio-demographic characteristics**. We used two socio-demographic characteristics reported by students, namely gender (1 = females, 2 = males) and nationality (1 = international, 2 = Dutch).

### 2.3. Statistical analyses

IBM SPSS Statistics Version 23 was used to assess descriptive statistics, bivariate correlations and attrition analyses of all variables. To assess attrition, we conducted a series of independent samples *t*-tests to examine potential differences in key variables (e.g., exhaustion, engagement, self-compassion, self-criticism and GPA) between participants who completed all three measurement points and those who did not. We did not find differences between retained students and those who dropped out across the three waves. As a result, we did not use imputation methods and opted for a complete case analysis. Full details of the attrition analyses, including t-values, p-values, and effect sizes for each variable, are reported in the supplementary materials.

In addition, AMOS 23 software was used to test longitudinal measurement invariance to ensure that the constructs were measured consistently over time. Following [88], we first established configural invariance, confirming that the same factor structure was consistent across time points. Next, we tested for metric (weak) invariance, where we constrained the factor loadings to be equal, ensuring that the relationships between observed variables and the latent construct remained the same. We then assessed scalar (strong) invariance, adding constraints on the intercepts to compare latent means across time. Lastly, we tested for strict invariance, ensuring that the residual variances were consistent across time points. The invariance steps were compared by evaluating the changes in the Comparative Fit Index (ΔCFI), as this metric is unaffected by sample size and model complexity [13]. A ΔCFI value of ≥ -0.01 suggests that the model fit does not significantly worsen with the addition of new constraints. Longitudinal invariance testing of measures is essential to draw valid conclusions about the developmental trajectories of self-compassion, self-criticism, engagement and exhaustion [cf. 86]. Subsequently, we continued with the main analyses. To examine the changes over time in exhaustion and engagement (*first research question*), we conducted separate unconditional linear growth models for each variable, assessing initial levels (intercepts), changes over time (slopes), and the covariances between intercepts and slopes. In these models, the intercept factor loadings were fixed at 1, while the slope factor loadings were fixed at 0, 0.5 and 1.

To examine the mediating role of engagement in the relationship between the trajectories of self-compassion and academic achievement, alongside the relationship between the trajectories of self-criticism and academic achievement and the mediating role of exhaustion (*second and third research questions*), two parallel process latent growth curve models (PP-LGCM) were used. In these two models, nationality and gender were included as covariates to account for their potential influence on the relationships examined. Furthermore, we fixed the time scores for the slope growth factor at 0, 1, and 2 and the coefficients of the intercept growth factor at 1 in the two models.

The statistical significance of indirect effects was tested using 5000 bootstrap samples and the 95% corrected confidence intervals (*Bc*CI) that did not cross zero were interpreted as a significant indirect effect. The PP-LGCM was analyzed in a structural equation modeling (SEM) framework [12] using the following model fit indices: chi-square statistics (χ^2^, *p* > .05), the root mean square error of approximation (RMSEA, criterion: less than 0.08), comparative fit index (CFI, criterion: greater than 0.90), Tucker- Lewis index (TLI, criterion: greater than 0.90) and the standardized root mean square residual (SRMR, criterion: less than 0.08) [33].

## 3. Results

### 3.1. Study population

The English and Dutch versions of questionnaires were initially sent to 600 medical students, of whom 334 filled them out at T1, 268 at T2 and 117 at T3. The faculty offers both a Dutch and English curriculum, which justified the inclusion of questionnaires in both languages to accommodate all participants. We only used complete data sets and no data imputation methods were applied. The final sample, consisting of 117 medical students, was characterized mostly by females (74.4%, M = 1.26, SD = .44) and Dutch students (78.6%, M = 1.79, SD = .41), which is representative of the broader demographics of medical students. The mean age of the participants was 18.38 (SD = 1.18).

### 3.2. Descriptive statistics and bivariate correlations

Descriptive statistics for the main study variables at the three measurement points are presented in Table 1 of the supplementary materials. The results show that self-compassion, engagement and exhaustion tend to decrease over the Bachelor’s studies. Conversely, self-criticism and academic achievement tend to increase over time. Table 1 of the supplementary materials also presents the correlations between the self-compassion, self-criticism, engagement, exhaustion and GPA for the three measurement points. Overall, the Pearson’s correlations are in the expected directions.

**Table 1:** Estimated mean, variance, covariance and overall model fit of the unconditional growth models.


ESTIMATES OF PARAMETERS	SELF-COMPASSION	SELF-CRITICISM	ENGAGEMENT	EXHAUSTION	GPA

Mean					

Intercept	3.45(.05)***	2.99(.08)***	5.32(.08)***	3.59(.09)***	6.81(.04)***

Slope	–.17(.07)*	.16(.07)*	–.43(.08)***	–.24(.11)*	.27(.04)***

Variance					

Intercept	.20(.05)***	.55(.10)***	.47(.09)***	.77(.16)***	.05(.03)

Slope	.15(.08)	.03(.10)	.29(.12)*	.65(.22)**	–.05(.05)

Covariance	–.01(.05)	.07(.08)	.06(.08)	–.10(.14)	–.01(.03)

Model fit	χ^2^ (3) = 2.9	χ^2^ (3) = .28	χ^2^ (3) = 2.7	χ^2^ (3) = 6.01	χ^2^ (2) = 10

	*p* > .05	*p* > .05	*p* > .05	*p* > .05	*p <* .01

	CFI = 1.00	CFI = 1.00	CFI = 1.00	CFI = .98	CFI = .90

	TLI = 1.00	TLI = 1.01	TLI = 1.00	TLI = .98	TLI = .85

	RMSEA = .00	RMSEA = .00	RMSEA = .00	RMSEA = .09	RMSEA = .18

	SRMR = .02	SRMR = .01	SRMR = .02	SRMR = .03	SRMR = .03


Note. *p < .05. **p < .01. ***p < .001.

### 3.3. Longitudinal measurement invariance

Self-compassion, self-criticism, engagement and exhaustion have to exhibit longitudinal measurement invariance to accurately assess their developmental trajectories in the unconditional latent growth models [86]. Results of the longitudinal measurement invariance are presented in Table 2 of the supplementary material and indicate that longitudinal measurement invariance was confirmed as the main index (ΔCFI) was ≥ –0.01 in each step of the invariance analysis, suggesting that the model fit did not significantly worsen with the addition of new constraints.

To conclude, longitudinal measurement invariance was confirmed for exhaustion, engagement, self-criticism and self-compassion, indicating that the constructs are measured consistently over time and that it is possible to compare the constructs between the three time points.

### 3.4. Developmental Changes Over Time

Unconditional latent growth models were performed to assess how the study variables change over time. Table 1 displays the goodness-of-fit indices for the estimated unconditional models, which were found to be acceptable. Additionally, Table 1 presents the unstandardized parameter estimates of these models. Specifically, it reports the average initial levels of the variables and the mean slopes.

The mean slopes indicate a steep decline over time for engagement, whereas the decrease in self-compassion and exhaustion is moderate. To categorize these changes, we followed an approach that considers the ratio between the slope coefficient and the standard deviation of the corresponding variable [see 15 for effect size interpretation]. Specifically, the ratio was –.45 for engagement, –.26 for self-compassion, and –.21 for exhaustion. Given that values around .20 are considered small, .50 moderate, and .80 large, engagement’s decline falls on the higher end of moderate, justifying the “steep” label, while self-compassion and exhaustion show more modest declines.

Moreover, the variances of the intercepts were significant for self-compassion, self-criticism, engagement, and exhaustion, suggesting systematic differences in initial levels among students. Regarding the slopes, significant variances were observed for engagement and exhaustion, indicating distinct individual growth trajectories for these variables. For self-compassion, the variance of the slope (0.15, *p* = .08) was marginally significant based on the conventional two-sided criterion (*p* < .05). However, since variances cannot be negative, one-sided testing is appropriate in this context [74], which makes the variance significant at the .05 level. This suggests that individual differences exist in how students’ self-compassion changed over time, even if this variation is less pronounced compared to engagement and exhaustion.

### 3.5. Associations Between Growth Trajectories

**Figure 1:**
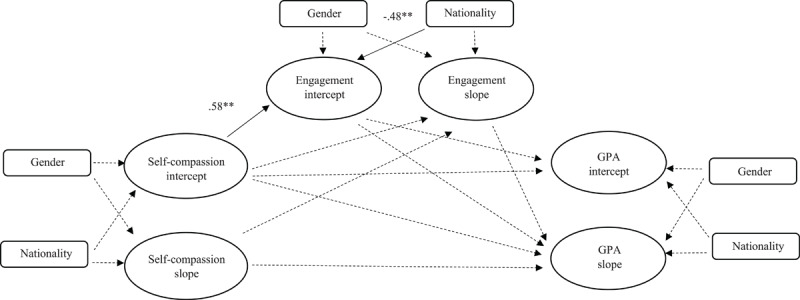
Latent growth model for the bright path: self-compassion, engagement, and GPA. Non-significant paths are denoted by dashed lines. Note. *p < .05. **p < .01. ***p < .001; nationality and gender were included as covariates in the ‘bright path’. All values are unstandardized.

We conducted two separate parallel process latent growth models to simultaneously assess the associations between the components of the growth trajectories for the research variables. Specifically, we examined the bright path, testing the associations between the trajectories of self-compassion and GPA and the mediating role of engagement. Additionally, we examined the dark path, investigating the association between the trajectories of self-criticism and GPA and the mediating role of exhaustion. The bright path showed acceptable fit indices: [χ^2^ = 50.26 (*df* = 32), *p* < .05, TLI = .91, CFI = .95, RMSEA = .07]. As can be seen in Figure 1, the only significant association was found on the intercept level (i.e., intercept-to-intercept). Specifically, we found that self-compassion was positively associated with engagement (β = 0.58, *p* < .01). Therefore, higher initial levels of self-compassion were associated with higher initial levels of engagement. Since this was the only significant path identified, it was not deemed necessary to further verify the mediating role of engagement.

In addition, nationality emerged as a significant covariate in the bright path. International students reported higher initial levels of engagement compared to Dutch students (intercept: β = –0.48, *p* < .01), suggesting that international students begin their studies with greater academic engagement.

**Figure 2:**
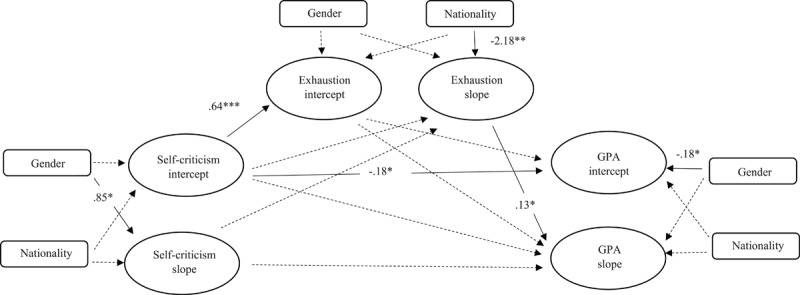
Latent growth model for the dark path: self-criticism, exhaustion, and GPA. Non-significant paths are denoted by dashed lines. Note. *p < .05. **p < .01. ***p < .001; nationality and gender were included as covariates in the ‘dark path’. All values are unstandardized.

For the dark path, the fit indices were acceptable: [χ^2^ = 53.41 (*df* = 32), *p* < .05, TLI = .91, CFI = .95, RMSEA = .07]. As depicted in Figure 2, at the intercept level (i.e., intercept-to-intercept), self-criticism showed a positive association with exhaustion (β = 0.64, *p* < .01), indicating that higher initial levels of self-criticism were linked to higher initial levels of exhaustion. Additionally, self-criticism was significantly and negatively correlated with GPA (β = –0.18, *p* < .05), suggesting that higher initial levels of self-criticism were associated with lower GPA. At the slope level (i.e., slope-to-slope), the only significant path identified was between exhaustion and GPA (β = 0.13, *p* < .05). This finding suggests that a decrease in exhaustion over time was positively associated with an increase in GPA throughout the Bachelor’s studies. In other words, students who experienced a reduction in exhaustion were more likely to achieve higher GPA over time.

In the dark path, we also tested the indirect relationships to identify the mediating effects of exhaustion. The results showed that exhaustion did not mediate the relationship between self-criticism and GPA.

Two significant covariate effects also emerged in the dark path. First, gender predicted the rate of change in self-criticism: males showed a steeper increase in self-criticism compared to females (slope: β = 0.85, *p* < .05). Second, nationality predicted the trajectory of exhaustion: Dutch students experienced a decline in exhaustion over time, whereas international students experienced an increase (slope: β = –2.18, *p* < .01).

## 4. Discussion

The first aim of this longitudinal study was to examine the changes over time of exhaustion and engagement among medical Bachelor students. Secondly, we examined two distinct pathways proposed by the JD-R model that impact well-being and performance, i.e., the motivational pathway and the health impairment pathway. The motivational path, or the ‘bright path’, was investigated by assessing the mediating role of engagement in the relationship between self-compassion and academic achievement.

The health impairment pathway, or the ‘dark path’, was examined by assessing the mediating role of exhaustion in the relationship between self-criticism and academic achievement. Our results revealed a trend of decreasing risk of exhaustion and a decline in engagement over the course of the Bachelor’s program. However, the growth trajectory for GPA was not well captured by a simple linear slope, which likely explains why the model fit indices for GPA did not meet conventional adequacy criteria. Therefore, the interpretation of GPA-related findings should be approached with caution, as the unconditional latent growth model for GPA did not demonstrate fully adequate fit.

Furthermore, our results did not support the hypothesised bright and dark pathways, as engagement did not mediate the relationship between self-compassion and academic achievement, and exhaustion did not mediate the relationship between self-criticism and GPA.

### 4.1. Temporal development of medical students’ engagement and exhaustion

The present study examined changes in exhaustion and engagement among students during their Bachelor’s program. Our findings indicate a moderate decline in exhaustion over time, which contrasts with previous research showing an increasing trend among medical students as they advance in their studies [30,39,66]. The differences in findings may be attributed to variations in institutional policy, academic environment, or methodological approaches used to assess the change in exhaustion over time.

Additionally, our results reveal a pronounced decline in engagement over time. Notably, students in our study began their program with relatively high engagement levels, exceeding those reported in previous research among medical students [1,37,47]. Given this initially high engagement, a decline over time may be expected as students adapt to academic routines and the novelty of their studies fades. This suggests that the downward trend may be a natural progression rather than being solely driven by external factors.

### 4.2. Navigating the bright and dark pathways of self-compassion and self-criticism

In this study, we explored whether engagement mediates the relationship between self-compassion and GPA (i.e., the bright path) and whether exhaustion mediates the relationship between self-criticism and GPA (i.e., the dark path), drawing on the JD-R framework. It is important to note that the interpretation of GPA-related findings should be approached with caution, given that the unconditional latent growth model for GPA did not demonstrate fully adequate fit.

Our findings indicate that neither engagement nor exhaustion serve as mediators in these relationships. One possible explanation lies in the scope of our model. Although we grounded our approach in the JD-R framework, we did not test the full model, which would have required incorporating study demands and resources. Indeed, the JD-R model posits that study demands and resources—such as the relationship between students and teachers, academic workload, institutional culture, and financial stressors [31]— interact with personal characteristics to influence engagement and burnout symptoms, ultimately shaping academic outcomes [4,89]. Previous research on university students supports the interaction between personal and study demands and resources, indicating that study demands mediate the relationship between personal demands and burnout symptoms [81], while study resources mediate the link between personal resources and engagement [80].

### 5. Implications

The findings of this study have several implications for educational institutions, university administrators, mental health professionals, and academic researchers. First, our findings suggest that exhaustion tends to decline over the course of the Bachelor’s program, which may indicate that students develop better coping mechanisms, adapt to academic demands, or benefit from institutional support systems over time. One possible explanation for the heightened exhaustion in the first year could be the challenges associated with transitioning to university life [76], coupled with binding study advice that may create additional pressure on students. Given this, universities could implement targeted interventions to support first-year students, such as mentoring programs, stress management workshops, and enhanced academic counselling [3,73]. Additionally, Dutch institutions might consider refining their study advice policies to ensure that they motivate students without adding undue pressure.

Second, the observed decline in engagement may indicate an adjustment from an initial period of high enthusiasm to a more stable academic routine. Given that students started with relatively high levels of engagement compared to previous studies [1,47], this decline could reflect a natural progression as the novelty of their studies fades. Universities may need to implement strategies to sustain motivation and interest throughout the program, ensuring that engagement remains consistent despite this adjustment. In this regard, research suggests that system-level reforms such as a transition to pass/fail grading [6,61,63,72], mindfulness training [17,84], and better communication between administration, faculty, and students [31] can foster university students’ well-being and engagement. Supporting students’ engagement also involves implementing collaborative and experiential activities, as well as developing institutional practices that create inclusive and stimulating environments. [43] emphasizes the importance of maintaining a stimulating intellectual environment, while [35] highlight the use of creative spaces to enhance engagement through novelty and interactivity. Additionally, fostering community through online discussion forums and utilizing technologies like virtual learning environments can further promote ongoing participation [25–26].

Third, our results indicate that focusing exclusively on personal resources and demands (i.e., self-compassion and self-criticism) is insufficient to fully understand the changes in the trajectories of engagement, exhaustion, and, consequently, academic achievement. This suggests that contextual factors should also be taken into account as, according to the JD-R model [4,89], they interact with personal resources and demands in predicting engagement, burnout symptoms and academic outcomes. The importance of contextual factors is supported by interventions conducted at the university level, where the academic environment was shown to have a significant impact on student mental health, engagement and academic achievement. For instance, environmental design strategies, such as the creation of sensory gardens [75] or the integration of indoor nature elements [77], have demonstrated how well-designed physical spaces can enhance mental health, engagement and academic achievement. Moreover, structural interventions, such as those fostering collaboration between leadership and healthcare departments [53], underscore the importance of organizational support in cultivating a healthier academic environment. On the other hand, interventions focused solely on enhancing personal resources such as self-compassion have proven less effective in promoting student mental health compared with the general population [for a meta-analysis, see 57].

Taken together, our results reinforce the need for a more integrated approach to enhancing engagement and academic outcomes while reducing the risk of experiencing exhaustion. Rather than focusing only on personal resources and demands, interventions should address contextual factors that can either hinder or support students’ engagement, mental health and academic success. A holistic approach that combines both personal and contextual elements is likely to be more effective in promoting well-being and achieving long-term positive outcomes.

## 6. Limitations and future directions

Some limitations should be considered. First, although a sample of at least 100 participants is adequate for latent growth models according to [29, p. 19], previous works reported that sample sizes should be not less than 200 at each time point [9 –10]. Therefore, our final sample of 117 participants may be relatively modest for testing LGCMs and mediation pathways. This limitation may have reduced the statistical power of our models, particularly for detecting smaller effects and indirect effects [71]. Consequently, non-significant mediation findings should be interpreted with caution, as they may reflect limited power rather than the absence of true effects. Future research should aim to recruit larger samples to increase the robustness, precision, and generalizability of findings, as well as to more reliably capture indirect effects. Second, the sample in our study is predominantly female and Dutch, which may limit the generalizability of our findings. Future research should aim for a more balanced sample with greater gender and ethnic diversity. Additionally, our limited sample size constrained testing of alternative models within the bright and dark pathways. Larger samples are needed for such analyses in future studies.

Further research is also needed to explain why, as identified in the current study, international students are at higher risk of experiencing exhaustion over time compared to their Dutch counterparts. Our findings contrast with previous research that found no significant differences in health-related constructs, such as psychological distress, depression, and anxiety, between international and domestic students [e.g., 41, 45]. This discrepancy highlights the importance for academic educators and curriculum designers to gain deeper insight into the diverse experiences of different student groups. Understanding why some groups may be more vulnerable to poor mental health in the university environment—while others may not—could help inform targeted interventions and support strategies.

Furthermore, we did not take into account the academic demands and resources experienced by medical students, despite evidence that these factors interact with personal demands and resources to influence engagement, burnout symptoms, and job outcomes [4,89]. Future research should include study demands and resources to better understand how they influence the trajectories of the variables included in the dark and bright pathways of the current study.

Another limitation of our study pertains to the nature of missing data, as participants who did not complete the questionnaires at one wave were not re-invited for subsequent waves. This approach may introduce bias and limit the ability to fully track changes over time. Although there was a relatively high dropout rate across waves, attrition analyses indicated no significant differences between participants who completed all waves and those who dropped out on key study variables. Thus, consistent with [16], while dropout can potentially affect representativeness, in the present study it did not introduce systematic bias. Future research should aim to invite all participants who started in the first wave to participate in subsequent waves, regardless of their questionnaire completion status in previous waves. This inclusive strategy would help mitigate potential biases and strengthen the robustness of the results.

While the present models emphasize a dark/bright dichotomy of negative and positive pathways, strictly separating these processes may oversimplify their interactions. In reality, positive pathways can buffer or modulate negative ones—for example, self-compassion may mitigate the effects of self-criticism. Future research could therefore examine the simultaneous contributions of self-compassion and self-criticism within both bright and dark paths, offering a more nuanced understanding of how these constructs interact to influence academic achievement.

## 7. Conclusion

The results of this study indicate that both engagement and exhaustion decline over the course of the Bachelor’s medical program. Additionally, we found no evidence that engagement mediates the relationship between self-compassion and GPA, nor that exhaustion mediates the relationship between self-criticism and GPA. Our study contributes to the existing literature by adopting a focus on engagement and exhaustion. By examining the changes in these constructs over time, our study addresses the calls for a more holistic perspective that accounts for positive and negative factors [1,23]. The current study emphasizes the importance of not just surviving in medical settings, but thriving within them. This finding underscores the need to foster environments where both well-being and engagement are prioritized, while also addressing negative factors, such as burnout symptoms, that can hinder student success. In addition, our findings suggest that, in line with the JD-R model, study demands and resources may play a crucial role in shaping engagement, burnout symptoms, and academic outcomes by interacting with personal demands and resources. This evidence reinforces the need for targeted interventions that address both institutional and individual factors to support student well-being and performance throughout their studies.

## Artificial Intelligence

This research did not utilize any artificial intelligence tools or technologies.

## Additional File

The additional file for this article can be found as follows:

10.5334/pme.1821.s1 Supplementary materials. Attrition analyses.
