# Supplementary material for: Dual Pathways From Self-Compassion and Self-Criticism to Academic Achievement: The Roles of Engagement and Exhaustion
Source: Perspect Med Educ. 2026 Feb 17;15(1):162–76. doi: 10.5334/pme.1821 (PMC12922675; doi:10.5334/pme.1821)
Supplement: Supplementary materials. — Attrition analyses. [file pme-15-1-1821-s1.pdf]

Supplementary materials

Attrition analyses

Attrition analyses were conducted to determine whether participants who completed all waves of data collection differed significantly from those who dropped out at any point. Participants who remained in the study across all waves (n = 117) were compared with those who did not complete all assessments (n = 257) on self-compassion, self-criticism, exhaustion, and engagement. The results showed no significant differences between retained and dropped-out participants in self-compassion ($t(243) = 0.32$, $p > .05$, d = .04), self-criticism ($t(243) = – 0.10$, $p > .05$, d = .01), exhaustion ($t(243) = 0.30$, $p > .05$, d = .03), and engagement ($t(243) = 0.83$, $p > .05$, d = .09). All effect sizes were negligible, indicating that attrition in the self-reported variables was random and did not introduce systematic bias into the results. Consequently, we opted to perform a complete case analysis (CCA), including only participants who completed all assessments at each measurement point, and no data imputation was applied. Finally, students' grade point averages (GPA) were obtained directly from university records and were available for all participants; therefore, no attrition analysis was required for this variable.

Table 1. Means, standard deviations and Pearson bivariate correlations among the study variables.

Dimension	M(SD)	1.	2.	3.	4.	5.	6.	7.	8.	9.	10.	11.	12.	13.	14.	15.
1. Self-compassion T1	3.47(.60)	--														
2. Self-compassion T2	3.33(.66)	.49***	--													
3. Self-compassion T3	3.30(.69)	.41***	.61***	--												
4. Self-criticism T1	3.01(.91)	-.34***	-.29***	-.24**	--											
5. Self-criticism T2	3.09(.97)	-.30***	-.49***	-.40***	67***	--										
6. Self-criticism T3	3.17(.99)	-.25**	-.39***	-.52***	.68***	.72***	--									
7. Engagement T1	5.32(.83)	.31***	.11	.17	-.26**	-.16	-.19*	--								
8. Engagement T2	5.10(.95)	.21*	.20*	.19*	-.17	-.10	-.11	.69***	--							
9. Engagement T3	4.90(1.06)	.23*	.09	.26**	-.22*	-.06	-.18	.58***	.70***	--						
10. Exhaustion T1	3.58(1.10)	-.19*	-.19*	-.11	.36***	.27**	.32***	-.21*	-.27**	-.27**	--					
11. Exhaustion T2	3.50(1.19)	-.09	-.35***	-.30***	.35***	.43***	.43***	-.16	-.31***	-.32***	.54***	--				
12. Exhaustion T3	3.33(1.23)	-.06	-.22*	-.33***	.26**	.29***	.41***	.01	-.12	-.28**	.45***	.72***	--			
13. GPA first year	6.83(.43)	.06	.08	.24*	-.08	-.25**	-.22*	-.02	-.07	.01	-.02	.01	-.08	---		
14. GPA second year	6.88(.50)	.03	.06	.18	.07	-.05	-.12	.01	-.05	.01	.04	.09	-.002	.60***	---	
15. GPA third year	7.13(.44)	.06	.20*	.13	-.07	-.19*	-.12	-.01	-.08	-.11	-.01	.12	.10	.41***	.44***	---

Note. *$p < .05$. **$p < .01$. ***$p < .001$

Table 2. Results of longitudinal measurement invariance across the three years of the Bachelor's program.

Model	χ^2 (*df*)	TLI	CFI	RMSEA	SRMR	Model comparison	$\Delta\chi^2$	Δdf	ΔCFI
Exhaustion									
M1. Configural invariance	42.86(15)	.9287	.9643	.0731	.0362	---	---	---	---
M2. Metric invariance	52.16(23)	.9513	.9627	.0604	.0415	M2-M1	9.3	8	-.002
M3. Scalar invariance	54.32(25)	.9550	.9625	.0580	.0598	M3-M2	2.16	2	.0001
M4. Strict invariance	73.99(35)	.9572	.9501	.0566	.0733	M4-M3	19.67	10	-.01
Engagement									
M1. Configural invariance	163.46(66)	.9020	.9401	.0651	.0486	---	---	---	---
M2. Metric invariance	170.03(74)	.9139	.9410	.0611	.0505	M2-M1	6.57	8	.0009
M3. Scalar invariance	176.30(76)	.9124	.9384	.0616	.0805	M3-M2	6.27	2	-.003
M4. Strict invariance	209.55(86)	.9046	.9241	.0643	.0822	M4-M3	33.25	10	-.01
Self-criticism									
M1. Configural invariance	25.87(24)	.9961	.9979	.0149	.0275	---	---	---	---
M2. Metric invariance	31.92(34)	1.00	1.00	.001	.0312	M2-M1	6.05	10	.002
M3. Scalar invariance	32.82(36)	1.00	1.00	.001	.0407	M3-M2	.90	2	.000
M4. Strict invariance	56.03(48)	.9917	.9911	.0219	.0592	M4-M3	23.21	12	-.001
Self-compassion									
M1. Configural invariance	40.77(21)	.8904	.9488	.0520	.0352	---	---	---	---
M2. Metric invariance	52.94(31)	.9176	.9432	.0451	.0454	M2-M1	12.17	10	-.001
M3. Scalar invariance	55.93(33)	.9191	.9407	.0447	.0613	M3-M2	2.99	2	-.002
M4. Strict invariance	73.25(49)	.9424	.9373	.0377	.0745	M4-M3	17.32	16	-.003
